# Combined detection of lymphocyte clonality and *MALT1* translocations in bronchoalveolar lavage fluid for diagnosing pulmonary lymphomas

**DOI:** 10.1038/s41598-021-02861-4

**Published:** 2021-12-06

**Authors:** Takashi Kido, Hiroshi Ishimoto, Hiroshi Ishii, Kanako Hara, Mutsumi Ozasa, Hiroki Kawabata, Toshinori Kawanami, Yu Suzuki, Hiroki Yoshikawa, Atsuko Hara, Noriho Sakamoto, Nobuhiro Matsumoto, Chiharu Yoshii, Junya Fukuoka, Masaki Fujita, Masamitsu Nakazato, Junichi Kadota, Hiroshi Mukae, Kazuhiro Yatera

**Affiliations:** 1 Department of Respiratory Medicine, University of Occupational and Environmental Health, Japan, Kitakyushu, Japan; 2 Department of Respiratory Medicine, Nagasaki University Graduate School of Biomedical Sciences, 1-7-1, Sakamoto, Nagasaki, 852-8501 Japan; 3 Department of Respiratory Medicine, Fukuoka University Hospital, Fukuoka, Japan; 4 Department of Pathology, Nagasaki University Graduate School of Biomedical Sciences, Nagasaki, Japan; 5 Department of Respiratory Medicine, Kokura Memorial Hospital, Kitakyushu, Japan; 6 Department of Respiratory Medicine and Infectious Disease, Faculty of Medicine, Oita University, Yufu, Japan; 7 Neurology, Respirology, Endocrinology and Metabolism, Internal Medicine, Faculty of Medicine, University of Miyazaki, Miyazaki, Japan; 8 Department of Respiratory Medicine, Wakamatsu Hospital of the University of Occupational and Environmental Health, Japan, Kitakyushu, Japan

**Keywords:** Cancer, Medical research, Molecular medicine, Oncology

## Abstract

Diagnosis of pulmonary lymphoma using small tissue samples is difficult and often requires surgical procedures; thus, a less invasive sampling method is desirable. We previously showed that pulmonary mucosa-associated lymphoid tissue (MALT) lymphoma can be diagnosed by detecting MALT lymphoma translocation gene 1 (*MALT1*) translocations in bronchoalveolar lavage fluid (BALF) cells. Analysis of B-cell clonality based on immunoglobulin heavy chain (*IGH*) gene rearrangements was also reportedly useful for diagnosing pulmonary lymphoma. The aim of this prospective multicenter study was to evaluate the yet unknown diagnostic potential of combined detection of *MALT1* translocations and clonality using BALF. We analyzed B- and T-cell clonality based on *IGH* and T-cell receptor (*TCR*) rearrangements together with *MALT1* translocations using BALF of patients with clinically suspected pulmonary lymphomas. In total, 39 patients were evaluated and categorized into three groups: B-cell lymphoma, lymphoproliferative disorders, and other diseases. *IGH* rearrangement detection for B-cell lymphoma diagnosis exhibited sensitivity and specificity of 88.9% and 90.0%, respectively. *TCR* rearrangements were not observed in patients with B-cell lymphomas. The presence of *IGH* rearrangements together with the absence of *TCR* rearrangements indicated 96.0% specificity for the diagnosis of B-cell lymphoma. The sensitivity and specificity of *MALT1* translocations for diagnosing MALT lymphoma were 28.6% and 100%, respectively. The combined detection of lymphocyte clonality and *MALT1* translocations using BALF is suitable for screening and diagnosis of B-cell lymphomas. Analysis of specific genes such as *MALT1* should improve the precision of B-cell lymphoma diagnosis.

## Introduction

Pulmonary lymphomas account for 0.5–1% of all pulmonary malignancies^1–3^. Among 1500 patients with lung cancer, 1.7% were diagnosed with primary pulmonary lymphoma and 2.7%—with secondary involvement of the lung by lymphoma^4^. Mucosa-associated lymphoid tissue (MALT) lymphoma is a low-grade B-cell malignancy representing the most frequently diagnosed type of pulmonary lymphomas^3,5^; other types include diffuse large B-cell lymphomas and T-cell lymphomas. Owing to their relative rarity and the difficulty associated with diagnosing this cancer, pulmonary lymphomas are challenging to manage, and their diagnostic strategy is not well established.

Minimally invasive techniques, such as bronchoscopy and computed tomography (CT)-guided lung biopsy, are often employed for diagnosing lung lesions to avoid more invasive surgical interventions^3^. However, tissue samples obtained via transbronchial lung biopsy (TBLB) or CT-guided lung biopsy are small and contain a mixture of infiltrates, including neoplastic lymphoid cells and other inflammatory cells^3^. These problems complicate the diagnosis of pulmonary lymphomas, necessitating diagnostic surgical interventions^1,3,4,6–8^ in 55.7–100% of patients^1,6,8–10^. Therefore, a less invasive diagnostic procedure is desirable.

Lymphocytic alveolitis is indicative of pulmonary lymphoma and can be detected by analyzing bronchoalveolar lavage fluid (BALF)^3,11^. In most lymphoproliferative disorders, T- and B-lymphocytes constitute > 90% and < 10% of the total BALF lymphocytes, respectively. In pulmonary B-cell lymphomas, bronchoalveolar B-lymphocyte levels increase to more than 10%^3,12–14^, and the clonality of B-lymphocytes, along with rearrangements of the immunoglobulin heavy chain-encoding *IGH* gene, may contribute to the diagnosis. Rearrangements of the T-cell receptor-encoding *TCR* gene have been reported to indicate T-cell lymphomas^15–19^; however, the diagnostic utility of analyzing this biomarker in BALF remains unclear. Rearrangements in the *MALT1* gene located on chromosome 18q21 and encoding MALT lymphoma translocation protein 1 are specific for MALT lymphomas; they include *IGH/MALT1* translocation and *API2/MALT1* fusion between the *API2* (apoptosis inhibitor 2) and *MALT1* genes^20,21^. We have previously shown that the detection of *MALT1* translocations in BALF cells by fluorescence in situ hybridization (FISH) is specific for pulmonary MALT lymphoma and could be used for diagnostic purposes^22^.

In this study, we analyzed the feasibility of using BALF for combined detection of lymphocyte clonality based on *IGH* and *TCR* rearrangements as well as *MALT1* translocations to diagnose pulmonary lymphomas.

## Methods

### Study design

This prospective multicenter cohort study was conducted in the University of Occupational and Environmental Health, Japan, Wakamatsu Hospital of University of Occupational and Environmental Health, Japan and five related hospitals (Nagasaki University Hospital, Fukuoka University Hospital, Kokura Kinen Hospital, Oita University Hospital, and University of Miyazaki Hospital). The study was performed according to the Declaration of Helsinki and approved by the Ethics Committee of Medical Research, University of Occupational and Environmental Health, Japan (Approval number: H25-109 and H27-094) and by each institutional review board (the Institutional Review Board at Nagasaki University Hospital, the Fukuoka University Hospital Institutional Review Board, the Ethics Committee of Kokura Memorial Hospital, the Ethics Committee of Oita University, and the Ethics Committees of Faculty of Medicine, University of Miyazaki). All adult participants provided written informed consent to participate in this study.

### Patients’ clinical and laboratory characteristics

Patients suspected of having pulmonary lymphoma by attending physicians based on their clinical history, laboratory data, and chest high-resolution CT (HRCT) results and admitted to our hospitals between October 2013 to March 2018 were enrolled. For all patients, the data regarding sex, age, smoking history, levels of serum lactate dehydrogenase (LDH) and soluble interleukin 2 receptor (sIL-2R), chest HRCT results, pathological findings, and final diagnosis were collected and analyzed. According to a previous report on the pattern-based classification of pulmonary lesions involved in MALT lymphoma^6^, lung lesion identified via chest HRCT were categorized into following types: (1) single nodule or solitary ground glass opacity, (2) multiple nodules or multiple and/or diffuse ground glass opacity, (3) single mass or single areas of airspace consolidation, (4) multiple mass or multiple areas of airspace consolidation. Lymphomas were diagnosed and classified according to the 2016 revision of the World Health Organization classification of lymphoid neoplasms^23^. Each patient’s final diagnosis was based on clinical, laboratory, and pathological evaluations, the opinions of pulmonologists, hematologists, radiologists, and pathologists, and the clinical course.

### BALF collection

BALF was obtained using flexible bronchofiberscopy for evaluation and diagnosis before treatment. After adequate local anesthesia with lidocaine, three 50 mL fractions of sterile saline were injected into the most extensively involved pulmonary segment determined by chest HRCT, gently retrieved using a suction syringe, and placed into sterile containers for microbiological and cytological examinations. BALF samples (30 mL) were stored at 4 °C until analysis of *IGH* and *TCR* rearrangements (15 mL) and *MALT1* translocations (15 mL). Patients from whom sufficient BALF volumes could not be recovered were excluded from the study.

### Detection of *IGH* and *TCR *rearrangements and *MALT1* translocations

**Figure 1:**
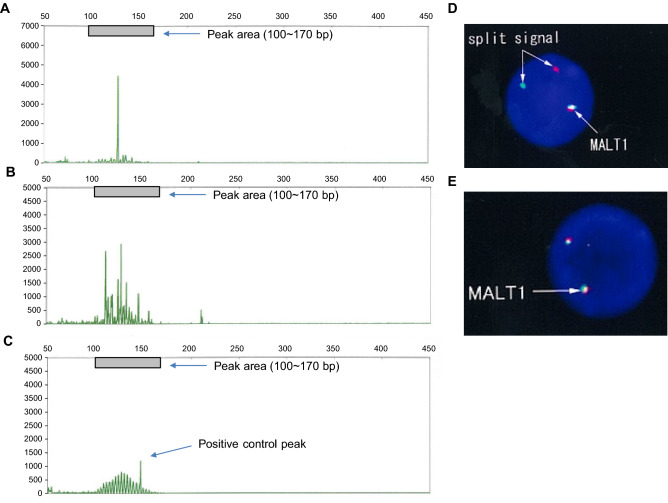
Examples of case analysis. (**A**,**B**) Positive results for the rearrangement of the VH(FR3)/JH region in the *IGH* gene of BALF lymphocytes. A peak higher than that of the positive control is detected on a low background (monoclonal pattern; **A**) or several distinct peaks are seen on a polyclonal background (oligoclonal pattern; **B**). (**C**) A peak for the positive control. (**D**,**E**) Results of *MALT1* translocation analysis by FISH. In case of *MALT1* translocations, the orange and green signals are separated (**D**), whereas in a normal cell they are fused (**E**).

*IGH* and *TCR* rearrangements in BALF lymphocytes were examined using IdentiClone^®^ IGH and TCRB Gene Clonality Assays (Invivoscribe Technologies, Inc., San Diego, CA, USA)^24,25^, which detect VH(FR1)/JH, VH(FR2)/JH, VH(FR3)/JH, DH1-6/JH, and DH7/JH regions in *IGH* and Vβ/Jβ1, Vβ/Jβ2, and Dβ/Jβ regions in *TCRB* by polymerase chain reaction (PCR) and capillary electrophoresis. When the electrophoresis pattern showed one distinct peak on a low background, which was higher than the peak of positive control (monoclonal pattern), the sample was considered positive, whereas samples with peaks lower than that of positive control or those with several higher peaks without individualization of one peak (oligoclonal pattern) were considered negative^13^ (Fig. 1). When the monoclonal pattern was observed in at least one of *IGH* or *TCR* regions, the gene was considered to have undergone rearrangement.

*MALT1* translocations were detected using FISH with the *MALT1* probe (Vysis MALT1 Break Apart FISH Probe Kit, Abbott Japan, Tokyo, Japan) consisting of a 460-kb 5′-end fragment labeled with SpectrumOrange and a 660-kb 3′-end fragment labeled with SpectrumGreen, as previously described^22,26–28^. Cells with MALT1 translocations would exhibit split orange and green signals (Fig. 1D), whereas normal cells would exhibit fused orange-green signals (Fig. 1E). Hybridized signals for each probe were evaluated in the interphase nuclei of 1,000 cells and positive cut-off values were set at 1.2% based on the analysis of blood samples from 30 healthy subjects performed by LSI Medience Corporation (Tokyo, Japan).

### Statistical analyses

The data are presented as the median (range) or the number of patients (%). All calculations were performed using the StatFlex software version 6 (Artech, Osaka, Japan). Continuous variables were compared by the Mann–Whitney *U*-test with Kruskal–Wallis test, and categorical variables—by chi-square or Fisher's exact tests as appropriate. *P* < 0.05 was considered to indicate statistical significance.

### Ethics approval and consent to participate

The study was performed in accordance with the Declaration of Helsinki and was approved by the Human and Animal Ethics Review Committee, of the University of Occupational and Environmental Health, Japan (Approval numbers: H25-109 and H27-094) and by each institutional review board. All adult participants provided written informed consent to participate in this study.

## Results

### Patients’ characteristics

**Figure 2:**
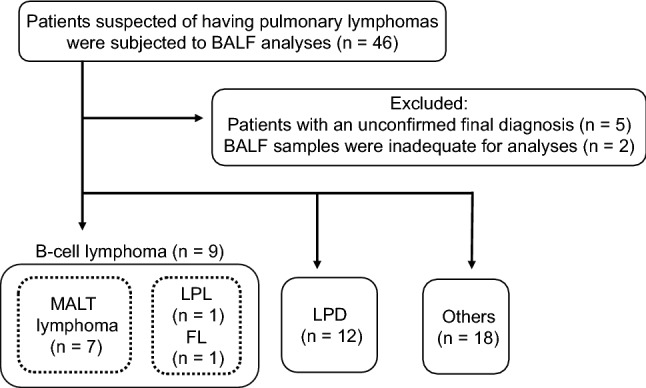
Flow chart of patient enrollment. *BALF* bronchoalveolar lavage fluid, *LPL* lymphoplasmacytic lymphoma, *FL* follicular lymphoma, *LPD* lymphoproliferative disorders, *MALT* mucosa-associated lymphoid tissue, *Others* other diseases.

In total, 46 patients were enrolled in the study; however, 5 patients with unconfirmed final diagnoses and 2 with BALF samples inadequate for molecular examination were excluded (Fig. 2). The 39 patients included for further analysis were divided into the B-cell lymphoma group (9 patients: 7 with MALT, 1 with lymphoplasmacytic, and 1 with follicular lymphomas), the lymphoproliferative disorder (LPD) group (12 patients: 5 with interstitial lung diseases due to Sjögren's syndrome, 4 with methotrexate (MTX)-related lymphoproliferative disorders, 2 with sarcoidosis, and 1 with multicentric Castleman’s disease), and the “Others” group (18 patients: 6 with infectious diseases, 5 with interstitial lung diseases, 3 with lung cancer, 1 with vasculitis, 1 with lung involvement in multiple myeloma, 1 with relapsing polychondritis, and 1 with granulomatous lung disease). None of the patients had T-cell lymphomas. The demographic and baseline characteristics of patients in each group are shown in Table 1. There were no significant differences among the groups in age, sex, smoking status, and serum levels of LDH and sIL-2R; median serum sIL-2R levels were higher than normal in all groups. There was no significant difference in chest HRCT findings among the groups in the ratio of hilar and/or mediastinal lymphadenopathy; however, the values of lung nodule and/or ground glass opacity were significantly lower, and the lung mass and/or airspace consolidation were significantly higher in the B-cell lymphoma group than in the LPD group. The median percentage of lymphocytes among BALF cells was increased (more than 10%) in all groups. TBLB was performed in 100%, 91.7%, and 83.3%, and surgical lung biopsy (SLB)—in 33.1%, 16.7%, and 5.5% of patients in the B-cell lymphoma, LPD, and other groups, respectively.

**Table 1:** Demographic and baseline characteristics of patients.

Characteristics	B-cell lymphoma (n = 9)	LPD (n = 12)	Others (n = 18)	*P-*value among all groups
Age, years (range)	72 (50–85)	63 (35–81)	69 (45–85)	0.4281
Men (%)	2 (22.2)	4 (33.3)	11 (61.1)	0.1091
Smokers (current or ex-smoker)	2 (22.2)	6 (50.0)	8 (44.4)	0.1297
**Serum levels**				
LDH, units/L (< 220)	207 (160–364)	231 (109–486)	206 (130–1564)	0.8941
sIL-2R, units/mL (< 500)	676 (268–5590)	776 (140–5117)	822 (258–11,700)	0.5968
**Chest HRCT findings**				
Lung nodule and/or ground glass opacity				
Solitary	1 (11.1)	1 (8.3)	1 (5.5)	0.8734
Multiple	2 (22.2)*	10 (83.3)	9 (50.0)	0.0190
Total	3 (33.3) ^‡^	11 (91.6)	10 (55.6)	0.0193
Lung mass and/or airspace consolidation				
Solitary	4 (44.4)	1 (8.3)	4 (22.2)	0.1502
Multiple	3 (33.3)	2 (16.7)	4 (22.2)	0.6641
Total	7 (77.7) ^§^	3 (25.0)	8 (44.4)	0.0488
Hilar and/or mediastinal lymphadenopathy	0 (0.0)	4 (36.4)	4 (21.1)	0.1683
Bronchoalveolar lavage				
Macrophages (%)	62.0 (38.0–88.4)	67.4 (20.9–86.3)	68.7 (0–96.0)	0.8614
Lymphocytes (%)	24.0 (8.3–40.0)	18.7 (8.3–76.5)	18.3 (3.0–40.0)	0.3428
Neutrophils (%)	4.8 (1.0–44.0)	2.0 (0.0–31.5)	7.3 (0.0–49.0)	0.2111
Eosinophils (%)	0.0 (0.0–3.0)	1.7 (0.0–12.6)	1.0 (0.0–29.0)	0.6291
Basophils (%)	0.0 (0.0–0.0)	0.0 (0.0–1.7)	0.0 (0.0–0.0)	0.3679
Lung biopsy				
TBLB	9 (100)	11 (91.7)	15 (83.3)	0.3906
CT-guided	0 (0.0)	0 (0.0)	1 (5.5)	0.5495
SLB	3 (33.3)	2 (16.7)	1 (5.5)	0.1671
Other biopsies				
Lip	1 (11.1)	2 (16.7)	0 (0.0)	0.2221
Bone	1 (11.1)	0 (0.0)	0 (0.0)	0.1808
TBNA	0 (0.0)	2 (16.7)	1 (5.5)	0.0933
Lymph node	2 (22.2)	3 (25.0)	2 (11.1)	0.5804
Subcutaneous nodule	0 (0.0)	1 (8.3)	0 (0.0)	0.3152

The data are presented as the median (range) or number (%).

*HRCT* high-resolution computer tomography, *LDH* lactate dehydrogenase, *LPD* lymphoproliferative disorder, *sIL-2R* soluble interleukin-2 receptor, *SLB* surgical lung biopsy, *TBLB* transbronchial lung biopsy, *TBNA* transbronchial needle aspiration.

*p = 0.0051, ^‡^*p* = 0.0050, and ^§^*p* = 0.0166 for B-cell lymphoma versus LPD.

### *IGH* and *TCR* rearrangements

**Figure 3:**
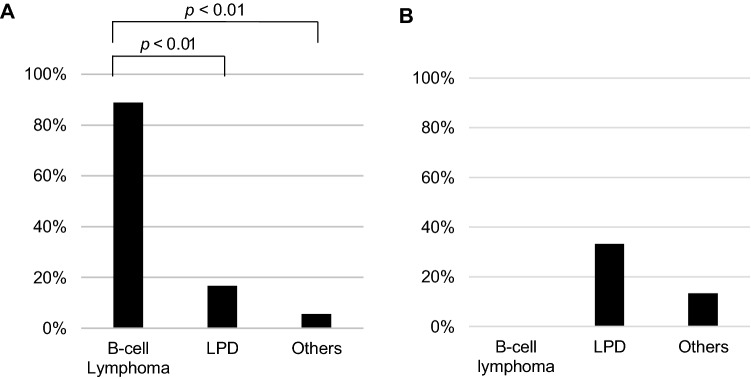
Clonality analysis in BALF*.* (**A**) Total *IGH* rearrangement rates (at least one positive region). (**B**) Total *TCR* rearrangement rates. *LPD* lymphoproliferative disorder.

The total rate of *IGH* rearrangement was significantly higher in the B-cell lymphoma group (88.9%) than in the LPD group (16.7%, *p* < 0.01) and the other group (5.5%, *p* < 0.01) (Fig. 3A). Rearrangements in specific *IGH* regions (VH(FR1)/JH, VH(FR2)/JH, VH(FR3)/JH, DH1-6/JH, and DH7/JH) are shown in Supplementary Figure S1 and Table S1. The sensitivity, specificity, and positive and negative predictive values of the total *IGH* rearrangement rate for the diagnosis of B-cell lymphoma were 88.9% 90.0%, 72.7%, and 96.4%, respectively.

*TCR* rearrangements in BALF cells were evaluated in 6 of 8, 9 of 12, and 15 of 18 patients with B-cell lymphoma, LPD, and other diseases, respectively, and the total rearrangement rates were calculated as 0.0%, 33.3% and 13.3%, respectively (Fig. 3B, Supplementary Figure S2 and Table S2). Although the differences between the groups were not statistically significant, the results indicated that all patients with B-cell lymphoma were negative for *TCR* rearrangements in BALF cells. The sensitivity, specificity, and positive and negative predictive values of the combination of *IGH* rearrangement-positive and *TCR* rearrangement-negative results for the diagnosis of B-cell lymphoma were 100%, 96.0%, 85.7%, and 100%, respectively.

### *MALT1* translocations

**Figure 4:**
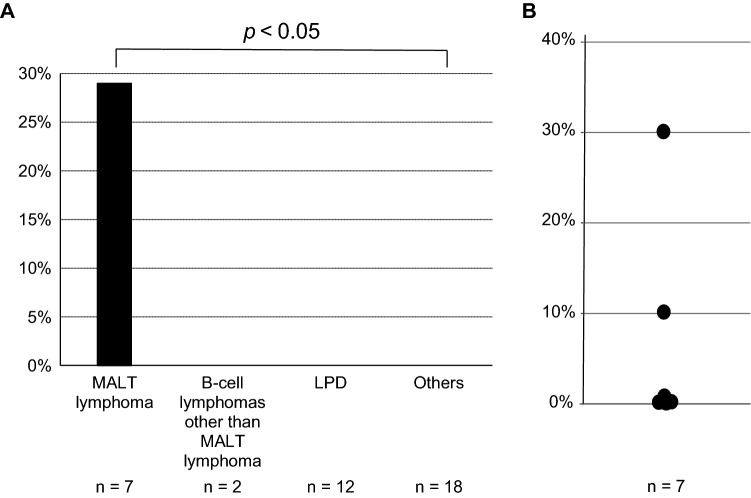
*MALT1* translocations in BALF cells. (**A**) *MALT1* translocation frequency in each patient group. (**B**) The percentage of *MALT1* translocation-positive lymphocytes among total BALF lymphocytes of patients with MALT lymphomas. *MALT*, mucosa-associated lymphoid tissue, *LPD* lymphoproliferative disorder.

Analysis of *MALT1* translocation frequencies in each group indicated that 28.6% (2/7) patients with MALT lymphoma were positive, whereas all other patients, including those with non-MALT B-cell lymphoma, were negative for *MALT1* translocation (Fig. 4A). The sensitivity and specificity of *MALT1* translocation detection for MALT lymphoma diagnosis were 28.6% and 100%, respectively. The rate of *MALT1* translocations among patients with MALT lymphoma was significantly higher than that in the other group (*p* < 0.05). The proportions of *MALT1* translocation-positive lymphocytes in BALF were 30.5% and 10.0% in the 2 positive patients (Fig. 4B) who also had *IGH* rearrangements (Table 2). These 2 patients, as well as 6 of 9 patients with B-cell lymphomas (66.7%), could be diagnosed without SLB.

**Table 2:** Procedures to diagnose B-cell lymphomas.

Lymphoma type	Biopsy type	Findings in BALF		
		*IGH* rearrangement	*TCR* rearrangement	*MALT1* translocation
MALT	TBLB	Positive	NA	Positive
MALT	TBLB	Positive	NA	Positive
MALT	TBLB, SLB	Positive	Negative	Negative
MALT	TBLB, SLB	Positive	Negative	Negative
MALT	TBLB, LN biopsy	Positive	Negative	Negative
MALT	TBLB, SLB	Positive	Negative	Negative
MALT	TBLB	Positive	Negative	Negative
Follicular	TBLB, bone biopsy	Positive	Negative	Negative
Lymphoplasmacytic	TBLB, LN biopsy	Negative	NA	Negative

*BALF* bronchoalveolar lavage fluid, *LN* lymph node, *SLB* surgical lung biopsy, *MALT* mucosa-associated lymphoid tissue, *IGH* immunoglobulin heavy chain-encoding gene, *TCR* T-cell receptor-encoding gene, *MALT1* MALT lymphoma translocation gene 1, *TBLB* transbronchial lung biopsy, *NA* not available.

## Discussion

This is the first study to analyze the diagnostic utility of *IGH* and *TCR* rearrangements in clonality analyses in addition to *MALT1* translocation in BALF of patients with clinically suspected pulmonary lymphoma. In our study, the sensitivity and specificity of *IGH* rearrangement results for B-cell lymphoma diagnosis were 88.9% and 90.0%, respectively. All patients with B-cell lymphoma were negative for *TCR* rearrangements, and the specificity of combined *IGH* rearrangement-positive and *TCR* rearrangement-negative results for the B-cell lymphomas diagnosis was 96.0%. The sensitivity and specificity of *MALT1* translocation testing for MALT lymphoma diagnosis were 28.6% and 100%, respectively. These findings suggest that the combined detection of *IGH* and *TCR* rearrangements in BALF cells is useful for the screening and diagnosis of B-cell lymphomas and that analysis of specific genes such as *MALT1* can improve diagnostic accuracy.

It is difficult to detect pulmonary lymphomas using small tissue samples obtained by TBLB or CT-guided lung biopsy, and 55.7–100% of patients need surgical interventions for definitive diagnosis^1,6,8–10^. In a retrospective review of 24 patients with pulmonary lymphomas, only 3 of 13 patients with pulmonary MALT lesions underwent complete surgical resection, whereas the others received chemotherapy^9^. In another retrospective study of 61 patients with pulmonary MALT lymphomas, no differences were observed in the time to progression between patients who underwent invasive surgical resection and those who received chemotherapy^8^. Thus, the development of molecular methods that enable diagnosis using small tissue samples is essential to help avoid invasive procedures. In the present study, 66.7% of patients with B-cell lymphomas were diagnosed without SLB, including 2 patients positive for both *IGH* rearrangements and *MALT1* translocations, indicating that the combined detection of these genetic aberrations can provide a more accurate diagnosis through a less invasive procedure.

The usefulness of detecting *IGH* rearrangements in BALF cells has already been assessed in several studies. Thus, it was shown that PCR analysis of *IGH* rearrangements in patients with B-cell pulmonary lymphomas was sensitive (6 of 7 patients) and specific (0 of 9 control individuals)^12^. Positive results were also obtained in 83% and 82% of patients with B-cell and MALT lymphomas at 90% and 97% specificity, respectively^13,14^. In the present study, we observed a similar sensitivity (88.9%) and specificity (90.0%) of *IGH* rearrangement detection for B-cell lymphoma diagnosis. We also analyzed *TCR* rearrangements using the *TCRB* clonal assay, which is considered a standard lymphoma diagnostic tool in Europe, detecting clonal *TCRB* rearrangements in 39.3–91.0% of T-cell lymphomas and in 3.8–16.0% of B-cell lymphomas^16–19^. In the present study, *TCR* rearrangements were absent in BALF cells of patients with B-cell lymphomas but present in 33.3% and 13.3% of patients with LPD and other diseases, respectively. Unfortunately, we did not analyze patients with T-cell pulmonary lymphoma because of its rarity. Our results revealed that the presence of *IGH* rearrangements combined with the absence of *TCR* rearrangements had 96% specificity for the diagnosis of B-cell lymphoma. Multiple analysis methods are employed in the diagnosis of lymphomas, such as clonality analyses, pathological findings, genetic profiles, and other clinical and laboratory findings. However, these tests are insufficient to confirm lymphoma and identify its subtype. Detection of *IGH* and *TCR* rearrangements may be a valuable diagnostic avenue for detection of pulmonary lymphoma. Unexpectedly, 75% of patients with MTX-related LPD exhibited clonal patterns (Supplementary Table S2), suggesting that *TCR* rearrangements may also be useful for diagnosing MTX-related LPD. Further studies are needed to evaluate the utility of *TCR* rearrangement testing in BALF cells for pulmonary T-cell lymphoma diagnosis.

Chromosomal translocations associated with MALT lymphoma include *API2/MALT1*, *IGH/MALT1*, *BCL10/IGH*, and trisomy 3 and 18^14,23,27^. The *API2/MALT1* translocation was detected in 30–70% of MALT lymphoma lung tissues obtained by SLB^20,21,27–29^, whereas the *IGH/MALT1* translocation was observed in 6–10% of patients with MALT lymphoma^27,28^. The probe for *MALT1* translocation used in the present study detected both *API2/MALT1* and *IGH/MALT1* translocations, and the *MALT1* translocation rate in patients with pulmonary MALT lymphomas observed here (28.6%) was consistent with previous reports^20,21,27–29^. Owing to its 100% specificity, the FISH-based detection of *MALT1* rearrangements in BALF cells would significantly improve the diagnostic accuracy for MALT lymphomas. Furthermore, the increased detection of genetic aberrations frequently observed in B-cell lymphomas, including *BCL2*, *BCL6*, *IGH,* and *MALT1* translocations, should promote the development of lymphoma gene panels for BALF in the era of next-generation sequencing.

Besides clonality and gene translocation testing, phenotyping using various markers such as cluster of differentiation (CD) can be employed to diagnose lymphomas^30^. In Europe, the detection of rearrangements in heavy and light immunoglobulin chain genes (*IGH*, *IGK,* and *IGL*) and *TCR* genes (*TCRB*, *TCRD*, and *TCRG*) by PCR is a standard approach used to diagnose B- and T-cell lymphomas^16,17,19,31^; however, the combination of these gene rearrangements in BALF cells has not been analyzed. The detection of both *IGH* rearrangements and CD expression in BALF cells has been shown to aid in diagnosing pulmonary B-cell lymphoma: it revealed B-cell clonality as well as an increase in B-cell (CD19- or CD20-positive) lymphocytes to over 10% of total BALF lymphocytes^3,12–14^. Collectively, these data suggest that the combined analysis of disease markers such as clonality, gene translocations, and CD expression in BALF should aid in diagnosing pulmonary lymphomas through a less invasive method.

## Conclusions

In conclusion, we evaluated the utility of the combined detection of clonality and *MALT1* translocations in BALF for the diagnosis of pulmonary lymphomas. Our results suggest that this combinatorial approach should help in identifying B-cell lymphomas through a less invasive method. Detection of additional genetic aberrations such as *MALT1* translocations in BALF cells can further specify the type of pulmonary lymphoma. Future studies should explore other combinatorial tools such as phenotyping along with *IGH*/*TCR* rearrangements to screen for pulmonary lymphomas.

## Supplementary Information

Supplementary Information 1.

Supplementary Information 2.

Supplementary Information 3.

## Data Availability

The data that support the findings of this study are available from the corresponding author upon reasonable request.

## Abbreviations

API2: Apoptosis inhibitor 2
BALF: Bronchoalveolar lavage fluid
CD: Cluster of differentiation
CT: Computed tomography
FISH: Fluorescence in situ hybridization
HRCT: High-resolution CT
IGH: Immunoglobulin heavy chain
LDH: Lactate dehydrogenase
LN: Lymph node
LPD: Lymphoproliferative disorder
MALT: Mucosa-associated lymphoid tissue
*MALT1*: Mucosa-associated lymphoid tissue lymphoma translocation gene 1
MTX: Methotrexate
sIL-2R: Soluble interleukin-2 receptor
SLB: Surgical lung biopsy
TBLB: Transbronchial lung biopsy
TCR: T-cell receptor
*TCRB*: TCR-beta gene
