# Supplementary figures and images for: Combined detection of lymphocyte clonality and MALT1 translocations in bronchoalveolar lavage fluid for diagnosing pulmonary lymphomas
Source: Sci Rep. 2021 Dec 6;11:23430. doi: 10.1038/s41598-021-02861-4 (PMC8648835; doi:10.1038/s41598-021-02861-4)

**A****VH(FR1)/JH**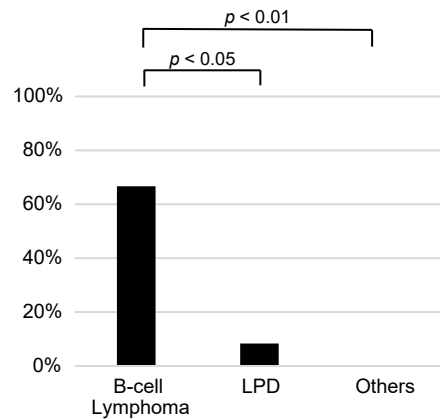**B****VH(FR2)/JH**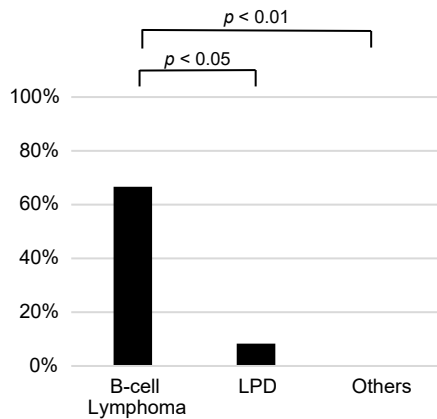**C****VH(FR3)/JH**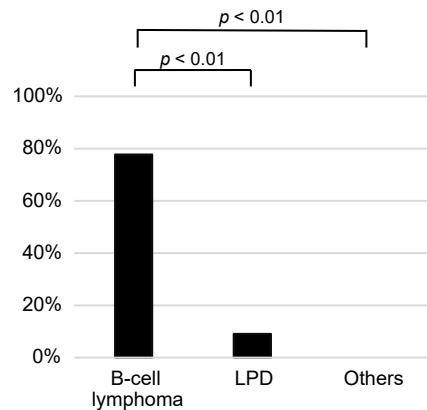**D****DH1-6/JH**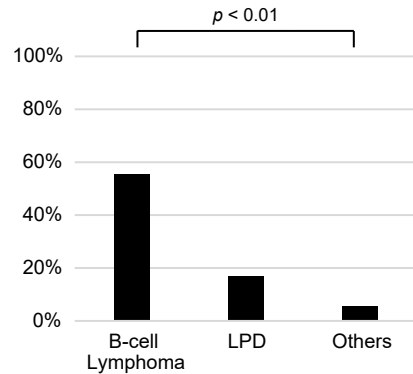**E****DH7/JH**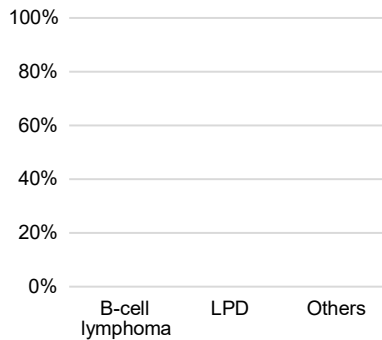

Supplement: Supplementary file 1 — Supplementary Information 1. [file 41598_2021_2861_MOESM1_ESM.pdf]

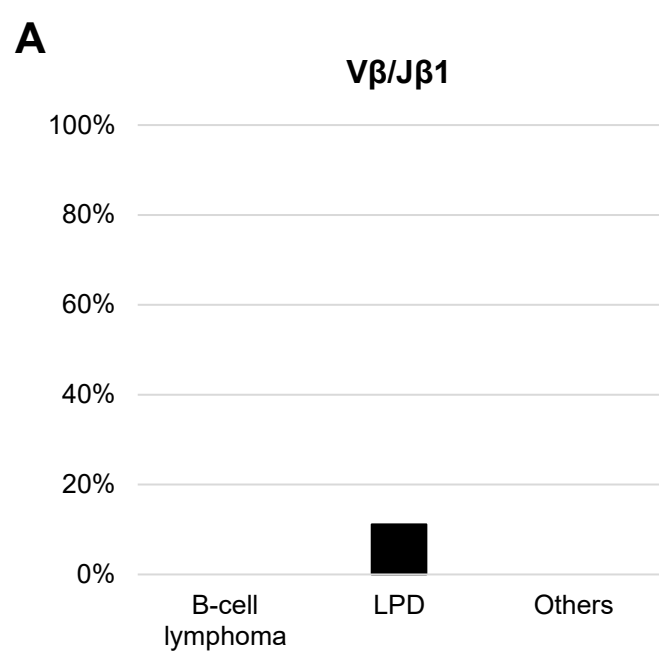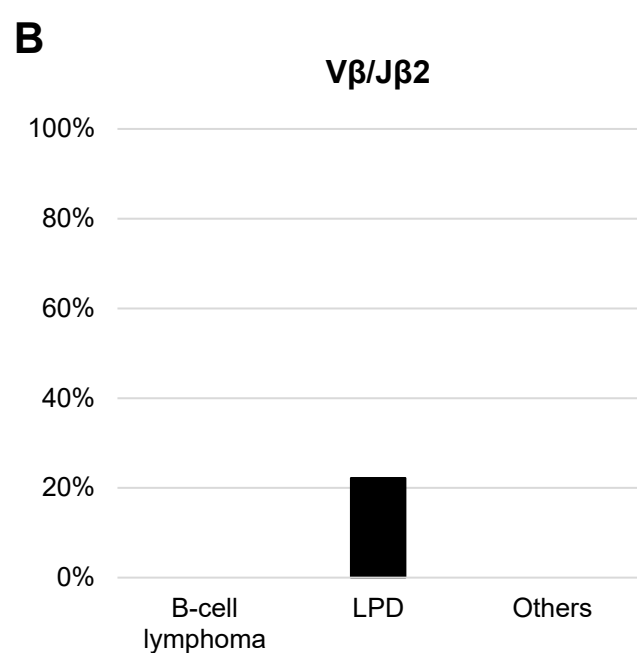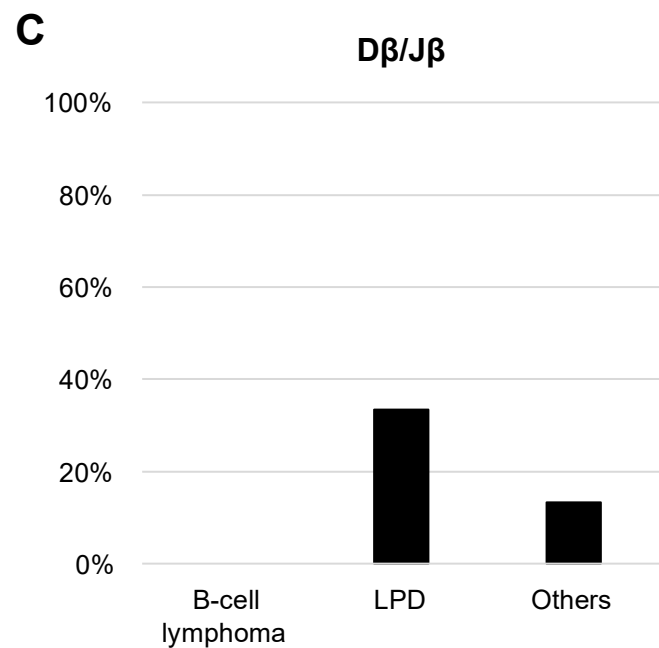

Supplement: Supplementary file 2 — Supplementary Information 2. [file 41598_2021_2861_MOESM2_ESM.pdf]
