# Supplementary material for: Combined detection of lymphocyte clonality and MALT1 translocations in bronchoalveolar lavage fluid for diagnosing pulmonary lymphomas
Source: Sci Rep. 2021 Dec 6;11:23430. doi: 10.1038/s41598-021-02861-4 (PMC8648835; doi:10.1038/s41598-021-02861-4)
Supplement: Supplementary file 3 — Supplementary Information 3. [file 41598_2021_2861_MOESM3_ESM.doc]

**Title:** Combined Detection of Lymphocyte Clonality and MALT1 Translocation in Bronchoalveolar Lavage Fluid for Diagnosing Pulmonary Lymphomas: A Multicenter Study of a Rare Disease Group

**Authors:**

Takashi Kido, ^1,2^ Hiroshi Ishimoto^1,2^, Hiroshi Ishii,^3^ Kanako Hara,^1^ Hiroki Kawabata,^1^ Toshinori Kawanami,^1^ Yu Suzuki,^4^ Hiroki Yoshikawa,^5^ Atsuko Hara,^1^ Noriho Sakamoto,^2^ Nobuhiro Matsumoto,^6^ Chiharu Yoshii,^7^ Masaki Fujita,^3^ Masamitsu Nakazato,^6^ Junichi Kadota,^5^ Hiroshi Mukae,^1,2^ Kazuhiro Yatera^1^

**Affiliations:**

^1^Department of Respiratory Medicine, University of Occupational and Environmental Health, Japan, Kitakyushu, Japan

^2^Department of Respiratory Medicine, Nagasaki University Graduate School of Biomedical Sciences, Nagasaki, Japan

^3^Department of Respiratory Medicine, Fukuoka University Hospital, Fukuoka, Japan

^4^Department of Respiratory Medicine, Kokura Memorial Hospital, Kitakyushu, Japan

^5^Department of Respiratory Medicine and Infectious disease, Oita University Faculty of Medicine, Yufu, Japan

^6^Neurology, Respirology, Endocrinology and Metabolism, Internal Medicine, Faculty of Medicine, University of Miyazaki, Miyazaki, Japan

^7^Department of Respiratory Medicine, Wakamatsu Hospital of the University of Occupational and Environmental Health, Japan, Kitakyushu, Japan

***Table S1.*** *Detailed information on IGH rearrangements in BALF* *lymphocytes*

| Group | Diagnosis | VH(FR1)/JH | VH(FR2)/JH | VH(FR3)/JH | DH1-6/JH | DH7/JH | Total |
| --- | --- | --- | --- | --- | --- | --- | --- |
| B-cell lymphoma | MALT lymphoma | * | + | + | + | - | + |
| B-cell lymphoma | MALT lymphoma | + | + | + | - | - | + |
| B-cell lymphoma | MALT lymphoma | + | + | + | - | - | + |
| B-cell lymphoma | MALT lymphoma | + | + | + | + | - | + |
| B-cell lymphoma | MALT lymphoma | + | + | + | + | - | + |
| B-cell lymphoma | MALT lymphoma | + | + | + | * | - | + |
| B-cell lymphoma | MALT lymphoma | + | - | + | + | - | + |
| B-cell lymphoma | Lymphoplasmacytic lymphoma | - | - | - | - | - | - |
| B-cell lymphoma | Follicular lymphoma | - | - | - | + | - | + |
| LPD | Sjögren's syndrome | - | - | - | - | - | - |
| LPD | Sjögren's syndrome | - | - | * | - | - | - |
| LPD | Sjögren's syndrome | - | - | * | - | - | - |
| LPD | Sjögren's syndrome | - | - | - | - | - | - |
| LPD | Sjögren's syndrome | - | - | - | - | - | - |
| LPD | MTX-related LPD | + | + | + | + | - | + |
| LPD | MTX-related LPD | - | - | - | - | - | - |
| LPD | MTX-related LPD | - | - | - | - | - | - |
| LPD | MTX-related LPD | - | - | - | - | - | - |
| LPD | Sarcoidosis | - | - | - | - | - | - |
| LPD | Sarcoidosis | - | - | - | + | - | + |
| LPD | multicentric Castleman’s disease | - | - | - | - | - | - |
| Others | Infectious disease | - | - | - | - | - | - |
| Others | Infectious disease | - | - | - | - | - | - |
| Others | Infectious disease | * | * | - | * | - | - |
| Others | Infectious disease | - | - | - | - | - | - |
| Others | Infectious disease | - | - | - | - | - | - |
| Others | Infectious disease | - | - | - | - | - | - |
| Others | Interstitial lung disease | - | - | - | + | - | + |
| Others | Interstitial lung disease | - | - | - | - | - | - |
| Others | Interstitial lung disease | - | - | - | - | - | - |
| Others | Interstitial lung disease | - | - | - | - | - | - |
| Others | Interstitial lung disease | - | - | - | - | - | - |
| Others | Lung cancer | - | - | - | - | - | - |
| Others | Lung cancer | - | - | - | - | - | - |
| Others | Lung cancer | - | - | - | - | - | - |
| Others | Granulomatous lung disease | - | - | - | - | - | - |
| Others | Lung involvement of multiple myeloma | - | - | - | - | - | - |
| Others | Relapsing polychondritis | - | - | - | - | - | - |
| Others | Vasculitis | - | - | - | - | - | - |

Abbreviations: BALF, bronchoalveolar lavage fluid; IGH, immunoglobulin heavy chain; LPD, lymphoproliferative disorder; MALT, mucosa-associated lymphoid tissue; MTX, methotrexate; Others, other diseases; +, clonal pattern; -, negative; *, oligoclonal pattern

***Table S2.*** *Detailed information on T-cell receptor rearrangements in BALF lymphocytes*

| Group | Diagnosis | VB/JB 1,2 | VB/JB2 | DB/JB | Total |
| --- | --- | --- | --- | --- | --- |
| B-cell lymphoma | MALT lymphoma | - | * | * | * |
| B-cell lymphoma | MALT lymphoma | - | - | - | - |
| B-cell lymphoma | MALT lymphoma | - | - | - | - |
| B-cell lymphoma | MALT lymphoma | - | * | - | * |
| B-cell lymphoma | MALT lymphoma | - | - | - | - |
| B-cell lymphoma | Follicular lymphoma | - | - | - | - |
| LPD | Sjögren's syndrome | * | * | * | * |
| LPD | Sjögren's syndrome | - | - | - | - |
| LPD | MTX-related LPD | * | * | + | + |
| LPD | MTX-related LPD | - | - | - | - |
| LPD | MTX-related LPD | + | + | + | + |
| LPD | MTX-related LPD | - | + | + | + |
| LPD | Sarcoidosis | - | - | - | - |
| LPD | Sarcoidosis | - | - | - | - |
| LPD | multicentric Castleman’s disease | - | * | - | * |
| Others | Infectious disease | - | * | + | + |
| Others | Infectious disease | - | * | - | * |
| Others | Infectious disease | - | - | + | + |
| Others | Infectious disease | - | - | - | - |
| Others | Infectious disease | - | * | - | * |
| Others | Interstitial lung disease | * | - | - | * |
| Others | Interstitial lung disease | - | - | - | - |
| Others | Interstitial lung disease | - | * | - | * |
| Others | Lung cancer | - | * | - | * |
| Others | Lung cancer | - | - | - | - |
| Others | Lung cancer | - | * | * | * |
| Others | Granulomatous lung disease | - | * | * | * |
| Others | Lung involvement of multiple myeloma | - | - | - | - |
| Others | Relapsing polychondritis | - | * | * | * |
| Others | Vasculitis | - | * | - | * |

Abbreviations: BALF, bronchoalveolar lavage fluid; LPD, lymphoproliferative disorders; MALT, mucosa-associated lymphoid tissue; MTX, methotrexate; Others, other diseases; +, clonal pattern; -, negative; *, oligoclonal pattern

**Figure S1.** Rearrangement rates for *IGH* gene regions in BALF lymphocytes*.* Detection rates (%) for VH(FR1)/JH (A), VH(FR2)/JH (B), VH(FR3)/JH (C), DH1-6/JH (D), and DH7/JH (E) in B-cell lymphoma, lymphoproliferative disorders (LPD), and other disease (Others) groups. BALF, bronchoalveolar lavage fluid; IGH, immunoglobulin heavy chain.

**Figure S2.** Rearrangement rates for *TCR* gene regions in BALF lymphocytes. Detection rates (%) for Vβ/Jβ1 (A), Vβ/Jβ2 (B), and Dβ/Jβ (C) in patients with B-cell lymphoma, lymphoproliferative disorders (LPD), and other diseases (Others). BALF, bronchoalveolar lavage fluid; TCR, T-cell receptor.
